# Novel Potassium Channels in Kidney Mitochondria: The Hyperpolarization-Activated and Cyclic Nucleotide-Gated HCN Channels

**DOI:** 10.3390/ijms20204995

**Published:** 2019-10-09

**Authors:** Daniel León-Aparicio, Carolina Salvador, Omar Emiliano Aparicio-Trejo, Alfredo Briones-Herrera, José Pedraza-Chaverri, Luis Vaca, Alicia Sampieri, Teresa Padilla-Flores, Zinaeli López-González, Juan C León-Contreras, Rogelio Hernández-Pando, Laura I Escobar

**Affiliations:** 1 Departamento de Fisiología, Facultad de Medicina, Universidad Nacional Autónoma de México (UNAM), Mexico City 04510, Mexico; dalebx4@gmail.com (D.L.-A.); caro.unam@gmail.com (C.S.); mhaytte@hotmail.com (T.P.-F.); zinaeli_lopezglz@hotmail.com (Z.L.-G.); 2 Departamento de Biología, Facultad de Química, Universidad Nacional Autónoma de México (UNAM), Mexico City 04510, Mexico; emilianoaparicio91@gmail.com (O.E.A.-T.); bhalfredo@gmail.com (A.B.-H.); pedraza@unam.mx (J.P.-C.); 3 Departamento de Biología Celular y del Desarrollo, Instituto de Fisiología Celular, Universidad Nacional Autónoma de México (UNAM), Mexico City 04510, Mexico; lvaca@ifc.unam.mx (L.V.); asampieri@ifc.unam.mx (A.S.); 4 Departamento de Patología, Instituto Nacional de Ciencias Médicas y Nutrición Salvador Zubirán, Mexico City 14080, Mexico; jcleonc@hotmail.com (J.C.L.-C.); rhdezpando@hotmail.com (R.H.-P.)

**Keywords:** mitochondria, kidney, mitochondrial potassium channel, hyperpolarization-activated cyclic nucleotide-gated cationic HCN channels in mitochondria, potassium transport in mitochondria, mitochondria uncoupling

## Abstract

Hyperpolarization-activated cationic HCN channels comprise four members (HCN1–4) that control dendritic integration, synaptic transmission and action potential firing. In the kidney, HCN1, HCN2 and HCN3 are differentially expressed and contribute to the transport of sodium, potassium (K^+^) and ammonium into the nephrons. HCN3 is regulated by K^+^ diets in the kidney. In this work we performed a proteomic analysis of HCN3 expressed in human embryonic kidney cells (HEK293 cells). More than 50% of the interacting proteins belonged to mitochondria. Therefore, we explored the presence of HCN channels in kidney mitochondria. By immunoblotting and immunogold electron microscopy HCN3 protein expression was found in rat kidney mitochondria; it was also confirmed in human kidney. Patch-clamp recordings of renal mitochondria and mitochondria from HEK293 cells overexpressing HCN1, HCN2 and HCN3 channels, stained with MitoTracker Green FM, indicated that only HCN3 could produce inwardly K^+^ currents that were inhibited by ZD7288, a specific blocker of HCN channels. Furthermore, ZD7288 caused inhibition of the oxygen consumption coupled to ATP synthesis and hyperpolarization of the inner mitochondrial membrane. In conclusion, we show for the first time that pacemaker HCN channels contribute to K^+^ transport in mitochondria facilitating the activity of the respiratory chain and ATP synthesis by controlling the inner mitochondrial membrane potential.

## 1. Introduction

The large number of mitochondria in the kidney provides the energy required to remove waste products and maintain electrolyte balance. Mitochondria are important organelles for cell viability, since they produce ATP via oxidative phosphorylation (OXPHOS) and play an active role in calcium homeostasis, reactive oxygen species (ROS) production and apoptosis. The electron transport system (ETS) and the ATP synthase are located in the inner mitochondrial membrane (IMM) and are essential components for the generation and preservation of energy metabolism [1]. The high negative value of the IMM potential (ΔΨm) required for oxidative phosphorylation is a powerful driving force for potassium (K^+^) uptake. Therefore, dynamic regulation of mitochondrial K^+^ flux in vivo is necessary to maintain the structural and functional membrane integrity for oxidative phosphorylation. To avoid swelling and lysis, the ion leakage in the mitochondria has to be balanced by extrusion of ions against the electrical gradient and this mechanism is accomplished by the mitochondrial K^+^ cycle [2].

The electrogenic transport of K^+^ into the mitochondrial matrix is strictly ion-channel-dependent. Activation of mitochondrial K^+^ channels is a hallmark of cell death and plays a central role in cardiomyocytes and neuron protection [3,4,5]. Mitochondrial K^+^ channels, such as the ATP-regulated (mitoKATP) channel, the Ca^2+^-activated K^+^ (mitoKCa) channel, the voltage-gated Kv1.3 (mitoKv1.3) channel and the two-pore domain TASK-3 (mitoTASK) channel, have been documented at the IMM of different organs, tissues or glioma cell lines. IMM K^+^ channels contribute to the regulation of the matrix volume, to establish ΔΨ and ΔpH, to calcium influx and to ROS production [6,7]. Activation of IMM K^+^ channels favors an uncoupling effect that could in turn decrease energetic efficiency [8]. So far only an ATP-sensitive K^+^ transport pathway has been documented in the rat kidney mitochondria [9].

The hyperpolarization-activated cyclic nucleotide-gated cationic (HCN) channels comprise four members termed HCN1–4 [10]; they have a pacemaker activity in excitable tissues controlling excitability, establishment of the resting membrane potential, dendritic integration, synaptic transmission and action potential firing [11,12]. Previously, we found that HCN channels transport ammonium (NH_4_^+^) in addition to Na^+^/K^+^ in the acid-secretory type-A intercalated cells of distal nephron segments of the kidney [13], contributing to the acid-base balance. Among HCN channel subtypes only HCN1, HCN2 and HCN3 are expressed and differentially distributed in the proximal convoluted tubule, the thick ascending limb of Henle, connecting tubule and collecting ducts of the rat kidney [14]. Due to the wide and abundant distribution of HCN3 in the kidney, in this work we performed a proteomic analysis of HCN3 overexpressed in HEK293 cells. Analysis of the proteomic data revealed interaction of HCN3 with mitochondria proteins. By immunoblotting, immunogold electron microscopy, patch-clamp recordings, oximetry and ΔΨ measurements, we confirmed this prediction and determined that HCN channels behave as open K^+^ channels in the kidney mitochondria, contributing to oxygen consumption, ATP synthesis and ΔΨ establishment.

## 2. Results

### 2.1. Proteomic Analysis of HCN3 Channel 

HCN3 is differentially expressed in the rat kidney: proximal tubules and thick ascending limb of Henle and its protein abundance is regulated by K^+^ metabolism [14]. In this study, proteomics and bioinformatics analyses were performed to establish a protein-protein interaction network (interactome) associated with HCN3. For this purpose, HCN3 protein was immunoprecipitated from transfected HEK293, electrophoretically separated and analyzed by mass spectrometry. Mitochondrial proteins were the most prominent protein family identified (Table 1).

### 2.2. Identification of HCN Potassium Channels in Kidney Mitochondria 

**Figure 1:**
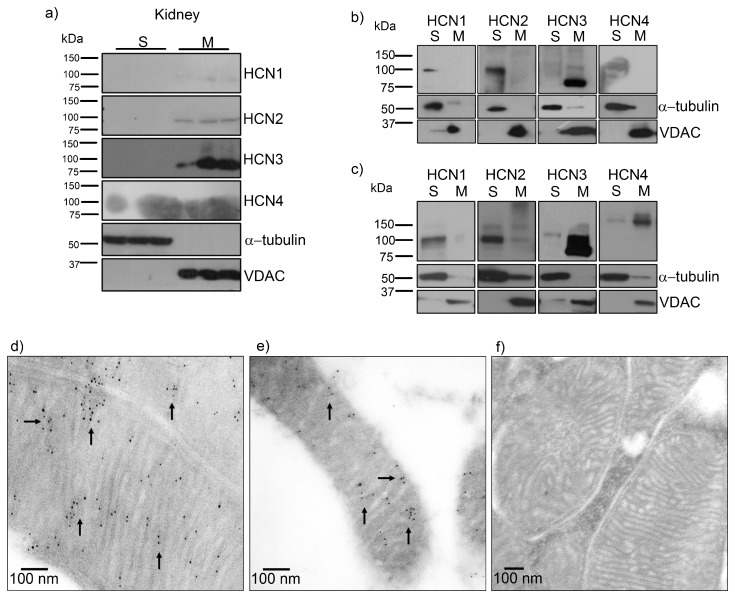
HCN3 channel expression in mitochondria isolated from renal cortex and HEK293 cells. (**a**) Immunoblotting of HCN1, HCN2, HCN3 and HCN4 with polyclonal antibodies detected only HCN2 (~94 kDa) and HCN3 (~86 kDa) in renal mitochondria. α-tubulin (~50 kDa) and voltage dependent anion channel (VDAC, ~31 kDa) were used as negative and positive controls of mitochondria abundance, respectively. (**b**) Immunoblotting of HCN1, HCN2, HCN3 and HCN4 in supernatant (S) and mitochondria (M) of control HEK293 cells. (**c**) Immunoblotting of HEK293 cells transfected with HCN1, HCN2, HCN3 and HCN4 in supernatant (S) and mitochondria (M). Only HCN3 (86 kDa) and HCN4 (160 kDa) were observed in HEK293 mitochondria. Immunogold electron microscopy localization of HCN3 (arrows) in the inner mitochondrial membrane of mitochondria from (**d**) rat (renal cortex) and (**e**) human kidney. (**f**) Negative control was performed in the absence of the primary antibody.

To investigate HCN3 expression in mitochondria, we performed immunoblot analysis with mitochondria isolated from the renal cortex. Immunoblotting of all HCN subtypes revealed very low amounts of HCN2 and abundant expression of HCN3 in kidney mitochondria. α-tubulin and VDAC were used as negative and positive controls of mitochondria abundance, respectively (Figure 1a). Therefore, we proceeded to study HCN3 protein expression in HEK293 cells. Before the transfection assay with each member of the HCN family (HCN1–4) in HEK293 ells, we analyzed and confirmed the expression of endogenous HCN1, HCN2 and HCN3 (Figure 1b). However, only HCN3 was detected in the mitochondrial fraction. In contrast, overexpression of HCN1–4 channels in HEK293 cells showed that HCN3 and HCN4 could be trafficked to mitochondria (Figure 1b,c).

HCN3 immunogold labeling was developed in rat and human renal tissue and examined by electron microscopy. HCN3 was localized specifically in the IMM in rat and human (Figure 1d,e). Human mitochondria looked smaller than rat mitochondria since they were recovered from formalin-fixed paraffin-embedded tissues.

### 2.3. Functional Characterization of HCN Channels in Mitochondria

**Figure 2:**
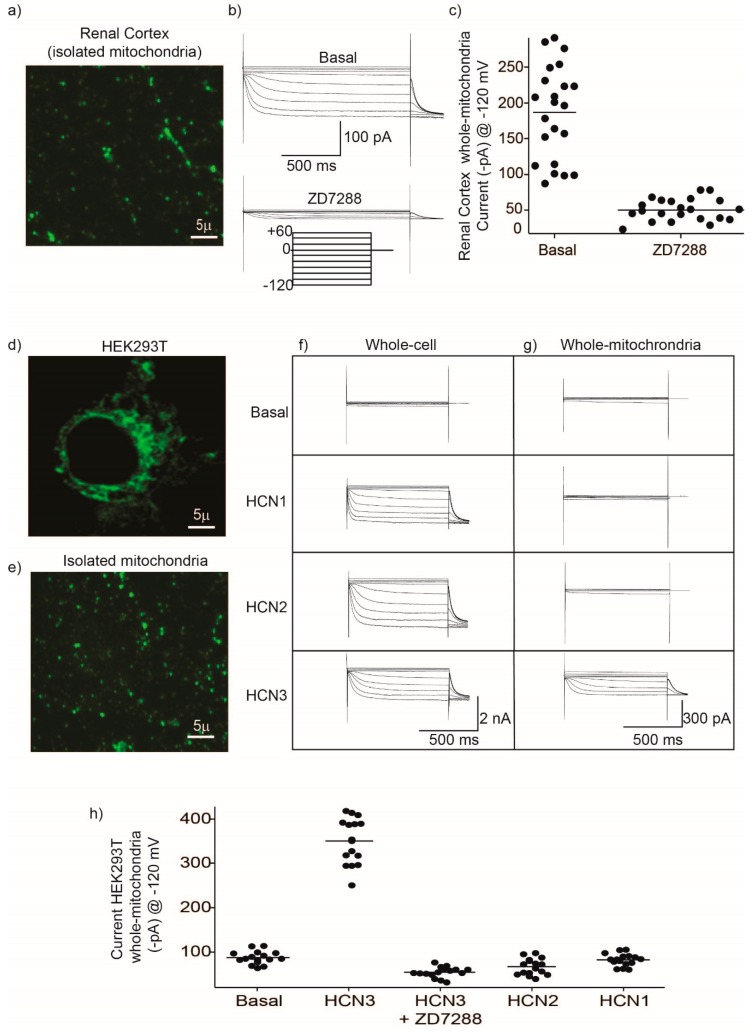
Inwardly potassium (K^+^) currents recorded in isolated mitochondria from the renal cortex and HEK293 cells correspond to HCN3. (**a**) Fluorescence microscopy imaging from isolated mitochondria obtained from the renal cortex. Mitochondria were incubated for 10 min with 5 µM of the fluorescent indicator MitoTracker Green FM. Excitation was 490 nm and emission collected at 525 nm. By fluorescence microscopy it was possible to separate mitochondria from debris during patch clamp experiments. (**b**) Whole-mitochondria voltage clamp currents were recorded with 20 mV steps from −120 mV to +60 mV and a holding potential of 0 mV. Basal shows a representative family of HCN currents obtained from a single mitochondrion and the same family of currents after 2 min of incubation with 50 µM of the inhibitor ZD7288. (**c**) Currents obtained at −120 mV from different independent mitochondria under basal conditions and after addition of ZD7288. Each filled circle represents a single measurement and the horizontal line indicates the mean value. (**d**) HEK293T cells labeled with the fluorescent indicator MitoTracker Green FM. (**e**) Isolated mitochondria from HEK293 cells labeled with MitoTracker Green FM. (**f**) Family of currents obtained in whole-cell configuration and (**g**) in whole-mitochondria, under basal conditions and from cells overexpressing HCN1, HCN2 and HCN3. (**h**) Currents recorded at −120 mV from mitochondria isolated from HEK293 control cells (Basal) and cells overexpressing HCN1, HCN2, HCN3 and HCN3 + ZD7288. Each filled circle represents a single measurement and the horizontal line indicates the mean value.

We aimed to study HCN3 functional expression in mitochondria isolated from the renal cortex (Figure 2a–c). To identify clearly mitochondria and separate them from cell debris, we incubated the isolated mitochondria with the mitochondria-selective fluorescent label MitoTracker Green FM (Figure 2a). Patch clamp recordings obtained from isolated mitochondria (whole-mitochondria recordings) evidenced the presence of hyperpolarization-activated K^+^ currents (Figure 2b,c) that were inhibited by the HCN-specific inhibitor, ZD7288 (Figure 2b,c). Based on immunoblotting assays (Figure 1a) and the time constant of activation (τ) at −100 mV of 1120 ± 450 ms (*n* = 17), the currents observed in kidney mitochondria were produced mostly by HCN3 channels (Figure 1a) since HCN2 displays a fast activation (τ = 562 ± 198) [15].

We proceeded next to explore the presence of HCN currents in HEK293 cells comparing whole-cell currents with currents from isolated mitochondria (whole-mitochondria recordings). Firstly, we labeled mitochondria with MitoTracker Green FM in intact living HEK293 cells (Figure 2d) and isolated mitochondria (Figure 2e). As illustrated in the Figure, MitoTracker Green FM labeled selectively mitochondria in culture cells and also in isolated mitochondria. Transfecting of all HCN1–3 plasmids resulted in whole-cell currents associated with each channel (Figure 2f). These results indicated that although each HCN channel subtype is expressed and localized at the plasma membrane of HEK293 cells (therefore identified by whole-cell recordings), only HCN3 produced functional currents in isolated mitochondria (Figure 2g,h).

### 2.4. ZD7288 Decreased the Mitochondrial Respiratory Control Index and ΔΨm

**Figure 3:**
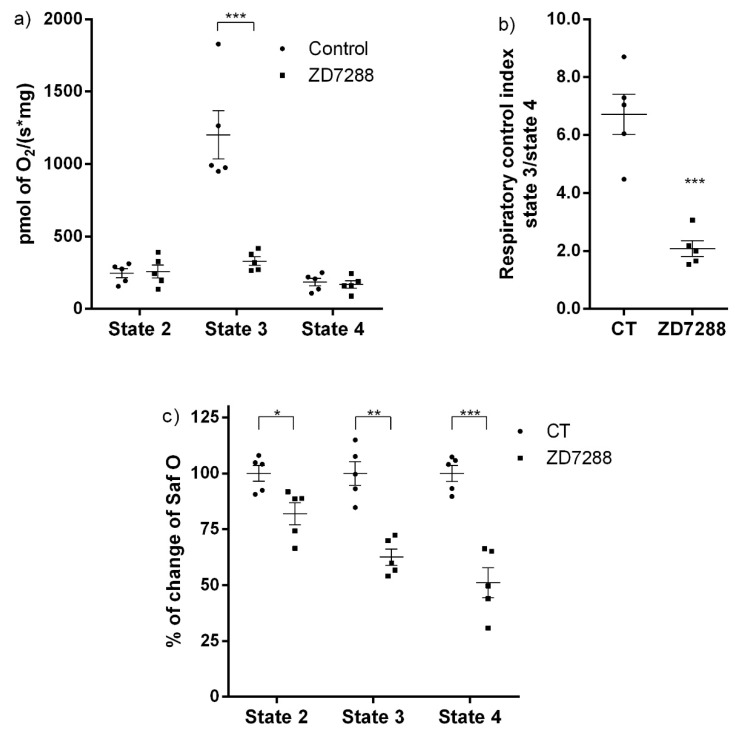
Effect of ZD7288 on the mitochondrial respiration rate and mitochondrial membrane potential (ΔΨm) on isolated mitochondria from renal cortex. (**a**) Respiratory parameters: state 2 (S2), state 3 (S3) and state 4 induced by oligomycin (S4o) and (**b**) respiratory control index (RCI). (**c**) Δψm in S2, S3 and S4o. Data are mean ± SEM, *n* = 5. *** *p* < 0.001, ** *p* < 0.01 and * *p* < 0.05.

To elucidate the functional role of HCN3 in mitochondria, the effect of ZD7288 was evaluated on isolated mitochondria from the renal cortex and HEK293 cells transfected with HCN3. It is well established that activation of NADH quinone oxidoreductase (Complex I) in respiratory state 2 (S2), increases hyperpolarization of the IMM by the electron transport chain proton pumping. Also, the addition of ADP or state 3 (S3) stimulates oxygen consumption due to ATP synthesis; this effect is inhibited by oligomycin (S4o). ZD7288 did not affect S2 and S4o. In contrast, ZD7288 drastically reduced oxygen consumption in S3 from 1202 ± 166.6 pmol O_2_ s^−1^ mg^−1^ to 330.4 ± 29.72 pmol O_2_ s^−1^ mg^−1^ (Figure 3a). As a consequence, mitochondrial coupling decreased: the respiratory control index (RCI) changed from 6.71 ± 0.70 to 2.09 ± 0.27 with ZD7288 (Figure 3b). Therefore, ZD7288 reduced the mitochondrial OXPHOS capacity. Interestingly, ΔΨm measured with safranin O diminished progressively from S2 to S3 to S4 with ZD7288. This effect was exacerbated in S3. Furthermore, ΔΨ in S4o also diminished with respect to the control group (Figure 3c). 

**Figure 4:**
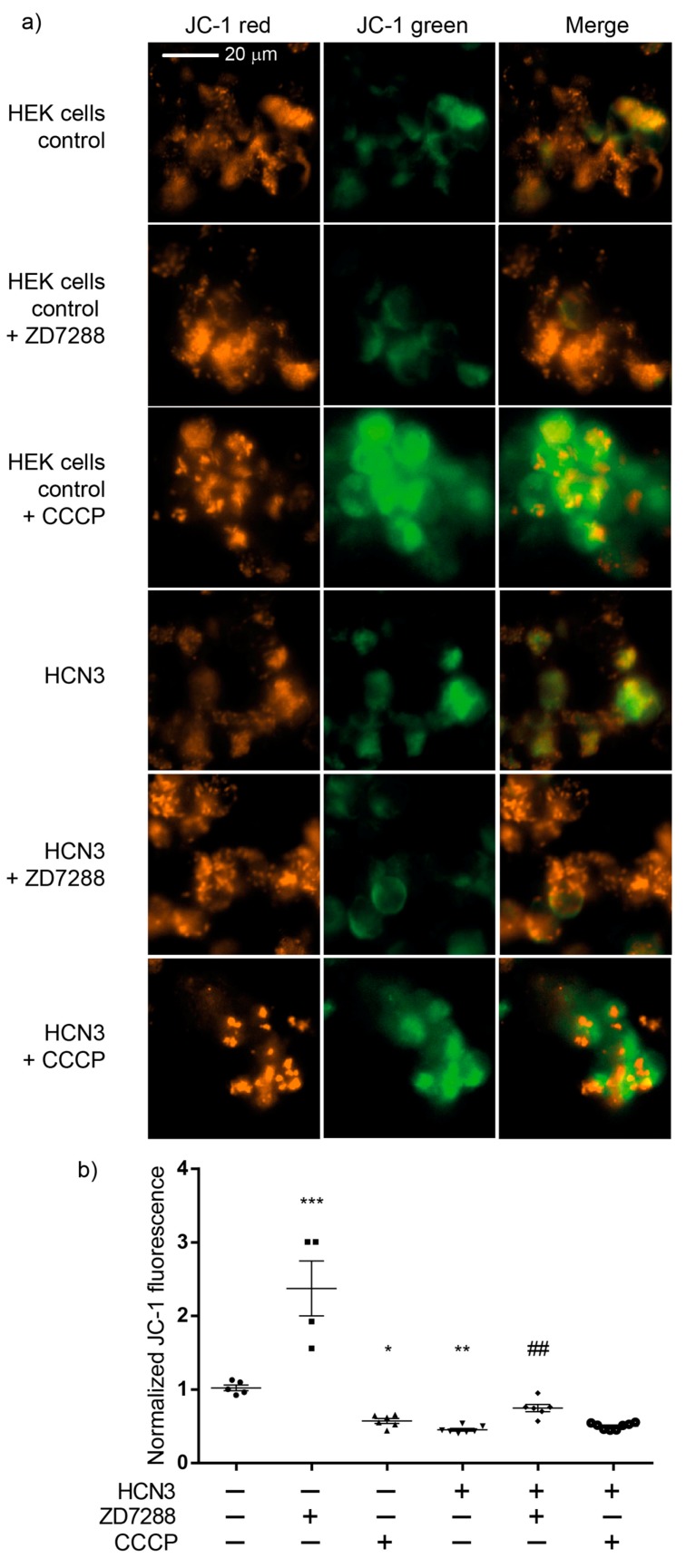
Effect of ZD7288 and carbonyl cyanide m-chlorophenylhydrazone (CCCP) on mitochondrial membrane potential (ΔΨm) in HEK293 cells (control) and HEK293 cells transfected with HCN3. (**a**) Visualization by Cytation 5 showing JC-1 red, JC-1 green and merge images of HEK293 cells. (**b**) The JC-1 fluorescence was quantified as the ratio of the red fluorescence (590 nm) to green fluorescence (525 nm) using Cytation 5 and the fluorescence was normalized with HEK293 cells (control) without inhibitor. ZD7288 (50 μM), CCCP (50 μM). Data are mean ± SEM, *n* = 4–8. *** *p* < 0.001, ** *p* < 0.01 and * *p* < 0.05 vs. HEK293 cells control. ^###^ *p* < 0.001 vs. HCN3 without inhibitor. JC-1 = 5,5’,6,6’-tetrachloro-1,1’,3,3’-tetraethylbenzimidazolylcarbocyanine iodide.

Cationic dyes preferentially accumulate in the mitochondrial matrix because of the higher ΔΨ (−120 to −160 mV) with respect to plasma membrane ΔΨ (−60 to −90 mV) [16]. To further estimate the contribution of HCN3 to ΔΨm, JC-1 assays were performed in control and transfected HEK293 cells with HCN3. As expected, ΔΨm was significantly lower in cells transfected with HCN3 than in control cells, which is consistent with the reduction in both routine and leak respiration observed in HEK293 transfected cells. Strikingly, addition of CCCP did not further diminish ΔΨ of the transfected cells, confirming the strong uncoupling effect of recombinant HCN3 K^+^ channel in the mitochondria (Figure 4a,b). 

Each merge image of Figure 4a corresponds to one representative experiment captured during four to eight independent assays performed for each experimental condition. Therefore, conclusions were extracted from the statistical analysis of the values plotted in Figure 4b. 

**Figure 5:**
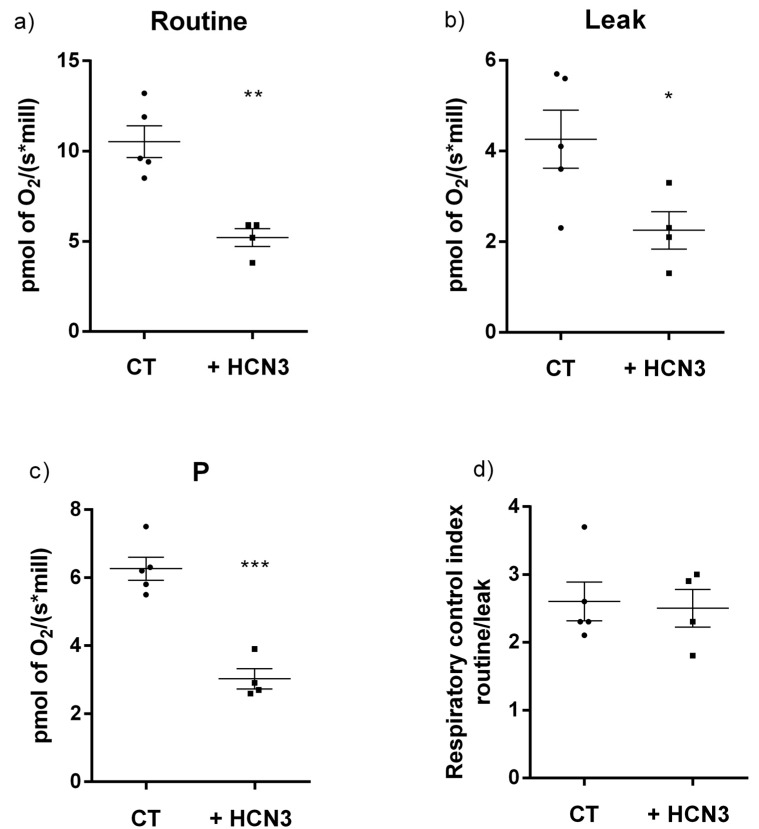
Respiratory parameters for control and HCN3 overexpressed in HEK293 cells. (**a**) Cellular routine respiration; (**b**) leak of respiration; (**c**) respiration associated to oxidative phosphorylation (P); (**d**) respiratory control index (RCI). Data is presented as mean ± SEM, *n* = 4–5. Unpaired *t*-test. *** *p* < 0.001, ** *p* < 0.01 and * *p* < 0.05 vs. control (CT).

It is well known that not all potential energy in mitochondria is transformed into ATP, as some processes like substrate transport and thermogenesis also depend on ΔΨm. Therefore, cell respiration in vitro is regulated by aerobic ATP demand but also by mitochondria processes that are required for cell growth, such as mitochondrial anabolism, and by processes that reduce mitochondrial coupling such as proton leak, electron leak and electron slip. So, cells incubated in the corresponding culture medium maintain this “basal (or routine) respiration”. The oxygen consumption of all mitochondrial processes that do not produce ATP can be evaluated by the oligomycin induction of the non-phosphorylating resting state (an uncoupled state) known as “leak respiration”. According to our results, endogenous HCN3 in kidney mitochondria and in mitochondria from HEK292 cells reduce ΔΨm. In fact, a stronger ΔΨm reduction effect was observed by overexpressing HCN3 in HEK293 cells (Figure 4b). Therefore, we performed an additional experiment in an attempt to explain the origin of this effect. In general, overexpression of HCN3 lowers “routine respiration” but also reduces “leak respiration” (Figure 5a,b). Although the value of respiration attributable to oxidative phosphorylation (P) decreased (Figure 5c), recombinant HCN3 did not change the respiratory control (RCI) (Figure 5d). Together these findings indicated that although mitochondrial ATP production decreased with channel overexpression, this was not due to a lower efficiency of mitochondrial function, but rather to a lower activity of all mitochondrial processes in the HCN3 group. A possible explanation could be a decrease in the mitochondrial mass since both groups produced ATP with equal efficiency (RCI did not change). In summary, the fact that both routine and leak respiration decreased with HCN3 overexpression seems to indicate fewer mitochondria. This hypothesis should be confirmed in the future.

## 3. Discussion

The kidney is, after the heart, the organ with the highest resting metabolic rates, mitochondrial content and oxygen consumption. The kidney contains abundant mitochondria to provide the energy necessary to recover or eliminate nutrients from the filtrated blood (amino acids, glucose, urea, electrolytes) and maintain the acid-base balance. Furthermore, healthy mitochondria are essential for the Na^+^-K^+^-ATPase activity and to generate the electrochemical gradients across the plasma membrane of epithelial cells. 

The development of quantitative high-throughput assays for identification of candidate proteins that interact with membrane proteins represents a powerful approach for biomarker discovery. In this work, targeted proteomics provided a sensitive assay to develop the hypothesis of the presence of the HCN3 channel in the kidney mitochondria.

The HCN family has been widely studied in the brain and heart, where they play a key role in the initiation and regulation of the electrical rhythmic activity [17,18]. In the mammalian heart and nervous system HCN channels contribute to spontaneous beating in pacemaker cells [17]. In nonpacing cells, inward cationic currents carried by HCN channels facilitate the establishment of the resting membrane properties by limiting the fluctuation of membrane potentials [18]. Although HCN channels form cationic non-selective channels, they display a higher selectivity for K^+^ over NH_4_^+^ and Na^+^. Our assays of ΔΨm helped to determine that HCN channels are K^+^ channels in the IMM and that their topology seems to be a mirror of the HCN channels in the plasma membrane but facing the cytosol instead of the extracellular side.

Previously we found that HCN channels are differentially distributed along the rat nephron. In particular HCN3 is observed near luminal membranes and the cytosol in the proximal convoluted tubule and towards basolateral membranes of the medullary thick ascending limb of Henle [14]. HCN3 has been found in the bladder and the junction of renal pelvis and has been implicated in ureteral peristaltic activity [19,20].

In this work we demonstrated for the first time and through several approaches that HCN channels, mostly HCN3, are located in mitochondria of the rat and human kidney by immunogold-labeling. Remarkably, by transfecting each HCN subtype in HEK293 cells, we observed that only HCN3 and HCN4 subtypes could be trafficked to mitochondria. In fact, we predicted these finding by proteomic analysis of HCN3 in HEK293 cells (Table 1). 

Mitochondria HCN3 functional expression was confirmed by patch-clamp recordings in rat kidney and HEK293 mitochondria. The magnitude of HCN inward K^+^ currents (100–300 pA) that were recorded by patch-clamp (Figure 2) should correspond to the electrical activity of several fused mitochondria. This experiment confirmed not only the presence of HCN K^+^ currents in the kidney mitochondria but, by HCN1–3 subtype transfection in HEK293T cells, that only HCN3 could be trafficked to these organelles. In the distal NH_2_ and COOH termini exist multiple regions specific to each of the four vertebrate HCN paralogs. These multiple regions of variable length and sequence confer isoform-specific properties. For example, in the COOH terminus of the HCN1, HCN2 and HCN4 isoforms, there is a block of residues conserved immediately downstream of the cyclic nucleotide binding domain [21,22]. Even when HCN3 subtype lacks this conserved block of residues, this would not explain HCN3 targeting to mitochondria since HCN4 is also trafficked to this organelle (Figure 1c). Further experiments should be performed to identify the targeting signals of HCN3 and HCN4 to mitochondria that have not been identified for other mammalian mitochondrial K^+^ channels. 

Since patch-clamp recordings of HCN channels in the whole mitochondria does not allow to distinguish between the outer and the IMM, we evaluated the effect of the HCN channel blocker ZD7288 on the mitochondrial oxygen consumption and ΔΨm on isolated renal mitochondria. Oxygen consumption by the respiratory chain in S2 or when ATP synthesis was inhibited with oligomycin in S4o was not affected by ZD7288. In contrast, oxygen consumption during ATP synthesis in S3 decreased to basal conditions with ZD7288. These results suggested that HCN3 K^+^ channel participates in the respiratory chain activity, especially during ATP synthesis. The effect of ZD7288 on the respiratory control index (RCI) confirmed this conclusion. It is important to underline that the control group is defined as the assay in the absence of ZD7288. States 2 and 4o (induced by oligomycin) must not be considered as basal respiration; these states are bioenergetic parameters determined according to the protocol followed in oxygraphic experiments with isolated mitochondria. Briefly, state 2 corresponds to the respiration due to the added substrates: sodium pyruvate, malate and glutamate in the absence of exogenous ADP. Later, ADP is added to achieve a stationary state of maximum oxygen flux triggered by oxidative phosphorylation (OXPHOS) or state 3. Finally, oligomycin is added to inhibit ATP synthase developing a non-phosphorylating stationary state (state 4o) when oxygen flux is maintained to compensate proton leak [1,3]. The difference between state 2 and state 4o is that in state 2 an ATPase residual activity may cause a recycling of ATP to ADP and, therefore, a residual activation of respiration. In contrast, in state 4o oligomycin inhibition of ATP synthase eliminates this contribution. Oxygen consumption in S3 is mostly determined by OXPHOS and depends highly on changes in ΔΨm [1]. Therefore, small changes in ΔΨm more severely affect the respiration rate in S3 than in S2 or S4o, where OXPHOS is not present. In conclusion, our results support that inhibition by ZD7288 affects OXPHOS more than it does leak states of respiration.

Regarding ΔΨm, changes in safranine O fluorescence were more sensitive to inhibition of HCN3 by ZD7288 than oximetry assays: safranine O fluorescence in states 2, 3 and 4o diminished by about 20%, 40% and 50%, respectively, with respect to basal conditions (Figure 3c). These results supported that HCN3 is an open K^+^ channel and its blockade by ZD7288 provoked a higher IMM polarization (i.e., a more negative ΔΨm). Therefore, HCN3 represents a K^+^ leak towards the mitochondrial matrix in resting conditions.

To further confirm the effect of HCN3 channel on the polarization of the IMM, assays were carried out with culture control HEK293T cells and HEK293T cells transfected with HCN3, using the lipophilic cationic dye JC-1. JC-1 forms aggregates with intense red fluorescence in healthy mitochondria with high ΔΨm (hyperpolarization) and monomers with green fluorescence when ΔΨm diminishes (depolarization). The higher the ratio of red to green fluorescence, the higher the polarization of the IMM [23]. The red to green fluorescence intensity ratio is not dependent on the mitochondrial size, density and shape. Therefore, JC-1 can be used both as qualitative and quantitative measure of ΔΨm. ZD7288 more than doubled JC-1 the fluorescence ratio with respect to control cells, supporting that endogenous expression of HCN3 enhances mitochondrial K^+^ fluxes with a mild uncoupling effect. Moreover, enrichment of mitochondria with HCN3 revealed a stronger uncoupling effect similar to that obtained with CCCP. In these circumstances, 50 µM ZD7288 reverses partially the uncoupling effect of overexpression of mitochondrial HCN3 since most probably not all HCN3 channels were blocked. 

The fact that recombinant HCN3 did not change the respiratory control (RCI) indicated that, although mitochondrial ATP production decreased with channel overexpression, this was not due to a lower efficiency of mitochondrial function. A decrement in the mitochondrial mass might be implied since mitochondria of both groups were equally efficient producing ATP. Furthermore, these results help to explain why ΔΨm is lower with HCN3 overexpression compared to ΔΨm in control HEK293 cells.

Only an ATP-sensitive K^+^ transport has been identified in kidney mitochondria [9]. In contrast to this ATP-K^+^ channel (KATP), which is inhibited by ATP, HCN channels are constitutively open in the mitochondria. Interestingly, voltage-gated mitochondrial K^+^ channels documented so far are closed in physiological conditions, for example: the maxi-conductance K^+^ channel (BKCa) and voltage-gated Kv1.3 channel. TWIK-related acid-sensitive K^+^ channel 2 (TASK3) regulates mitochondrial morphology, membrane potential and aldosterone production in adrenal cortex [24]. All K^+^ channels have been involved in the regulation of mitochondrial respiration, ΔΨm and ROS production within mitochondria. Opening of mitochondrial K^+^ channels has been associated with cytoprotection, while closed K^+^ channels may facilitate cell death [6,7]. The IMM K^+^ channels identified so far comprise BKCa [25], intermediate-conductance K^+^ channel (IKCa) [26], small-conductance K^+^ channel (SKCa) [27], Kv1.3 [28], KATP channel [29], two-pore TASK-3 [30], Kv1.5 [31], ROMK2 and Kv7.4 [32].

The proton pumps of the respiratory chain maintain ΔΨm in the range of −150 to −180 mV in the IMM. While Δp (combination of both ΔΨm and the mitochondrial pH gradient, ΔpH_m_) provides the bioenergetic driving force for and regulates ATP production, ΔΨm provides the charge gradient required for mitochondrial Ca^2+^ sequestration and production of reactive oxygen species (ROS) [33]. The opening of K^+^ channels provokes depolarization (uncoupling effect) while their blocking induces hyperpolarization in the IMM [34]. 

The mitochondrial electrochemical equilibrium is continuously maintained by the coupling of K^+^ channels and K^+^ efflux mediated by exchangers [35]. Mitochondrial swelling occurs after continuous K^+^ entry, caused by osmotic entrance of water through aquaporins and triggers the blockade of mitochondrial functions, favoring the release of cytochrome c. To avoid osmotic volume changes and mitochondrial depolarization, the positive K^+^ influx is counterbalanced by the efflux of protons (H^+^) through the activities of the respiratory chain complexes and by the K^+^/H^+^ antiporter (i.e., K^+^ “futile cycle”) [36]. 

## 4. Materials and Methods

### 4.1. Reactives, Antibodies and Plasmids

Antimycin A, adenosine diphosphate (ADP), (4-(2-hydroxyethyl)-1-piperazineethanesulfonic acid) (HEPES), fat-free bovine serum albumin (BSA), carbonyl cyanide m-chlorophenylhydrazone (CCCP), D-sucrose, ethylenediaminetetraacetic acid (EDTA), ethylene glycol-bis(2-aminoethylether)-N,N,N′,N′-tetraacetic acid (EGTA), K-lactobionate acid, mannitol, magnesium chloride (MgCl_2_) tetrahydrate, oligomycin, rotenone, safranin O, sodium phosphate dibasic (Na_2_HPO_4_), sodium phosphate monobasic (NaH_2_PO_4_), sodium glutamate, sodium malate, sodium pyruvate, taurine, polylysine, mannitol and anti-α-tubulin (t9026) were purchased from Sigma-Aldrich (St. Louis, MO, USA). Dulbecco’s Modified Eagle Medium (DMEM, 31600-083) Optimem (22600-134) were purchased from Gibco, Life Technologies. Dynabeads protein G (10003D), MitoTracker Green FM, NuPAGE^®^ LDS buffer 4×, NuPAGE (NP0007) 10% Bis-Tris protein gels, MOPS sodium dodecyl sulfate (SDS) buffer, 5,5’,6,6’-tetrachloro-1,1’,3,3’-tetraethylbenzimidazolylcarbocyanine iodide (JC-1, T3168), anti-green fluorescent protein (GFP, clone 3E6) HindIII and kpnI were purchased from Invitrogen by Thermo Fisher Scientific. Fetal Bovine Serum was purchased from Biowest (S1650). Normal mouse IgG (sc-2025) and polyethyleneimine (PEI, sc360988A) were purchased from Santa Cruz Biotechnology (Dallas, TX, USA). Bio-safe Coomassie G250 was purchased from Bio-Rad (Hercules, CA, USA). Polyclonal voltage-dependent anion channel (VDAC, ab34726) was purchased from Abcam (Cambridge, UK) and Immobilon Western Chemiluminescent horseradish peroxidase (HRP) substrate was from Millipore (Mexico City, México). Anti-HCN1, anti-HCN2, anti-HCN3 and anti-HCN4 were purchased from Alomone (Jerusalem, Israel). cOmplete™ Protease Inhibitor Cocktail Roche, Mexico. ZD7288 was purchased from Tocris (Minneapolis, MN, USA). pcDNA3-mHCN1, pcDNA3-mHCN2, pcDNA3-hHCN4 and pEGFP·C3 were provided by Luis Vaca. pcDNA3-mHCN3 was provided by Andreas Ludwig.

### 4.2. DNA Constructs

pcDNA3-mHCN3 was subcloned into eukaryotic expression vector pEGFP∙C3 using HindIII and KpnI. After subcloning, the sequence was confirmed by restriction assay and DNA sequencing analysis using an automated DNA sequencing technique.

### 4.3. Cultures and Transfection of HEK293 Cells

Human embryonic kidney cells (HEK293T cells) were grown in DMEM supplemented with 100 U/mL penicillin, 100 mg/mL streptomycin and 10% fetal bovine serum at 37 °C in a humidified atmosphere with 5% CO_2_. HEK293T cells were grown in tissue culture and transfected at 80% confluence using 1 mg/mL PEI and Optimem. Briefly, 1 µg of each plasmid pcDNA3-mHCN1, pcDNA3-mHCN2, pcDNA3-mHCN3 and pcDNA3-hHCN4 were diluted in low serum medium Optimem and mixed with the transfection reagent PEI, incubated at room temperature for 20–25 min and added to cultures. HEK293 cells were washed with cold phosphate buffer saline (PBS). For IP, 5 µg of GFP-HCN3 was transfected in HEK cells. 

### 4.4. Proteomic Assays

For GFP-HCN3 immunoprecipitation from HEK293T cells lysate, 5 µg of anti-GFP and 50 µL of Dynabeads protein G were mixed and incubated at 4 °C for 2 h. Then, 2 mg/mL of sample was added to the antibody-beads complex and incubated at 4 °C for 2 h. As control we used 2 µg/mL normal mouse IgG. Sample was extensively washed with radioimmunoprecipitation (RIPA) buffer with Protease Inhibitor Cocktail. NuPAGE LDS buffer was added and boiled for 7 min to separate the beads. The supernatant (30 µL) was loaded on NuPAGE 10% Bis-Tris protein gels in MOPS SDS buffer. Electrophoresis was run at 150 V for 20 min and the gel was stained with Bio-safe Coomassie G250. Samples from gel were enzymatically digested according to the modified protocol from [37]. Then, 4 µL per sample were loaded into Symmetry C18 Trap V/M precolumn (Waters, MA, USA); 180 μm × 20 mm, 100 Å pore size, 5 μm particle size desalted using as a mobile phase A, 0.1% formic acid (FA) in H_2_O; and mobile phase B, 0.1% FA in acetonitrile; under the followed isocratic gradient: 99.9% mobile phase A and 0.1% of mobile phase B at a flow of 5 μL min^-1^ for 3 min. After, peptides were loaded and separated on a HSS T3 C18 Column 75 μm × 150 mm (Waters); 100 Å pore size, 1.8 μm particle size; using an UPLC ACQUITY M-Class (Waters) with the same mobile phases under the following gradient: 0 min 7% B, 30.37 min 40% B, 32.03–35.34 min 85% B, 37–47 min 7% B at a flow of 400 nL min^−1^ and 45 °C. The spectral data were acquired in a mass spectrometer with electrospray ionization (ESI) and ion mobility separation (IMS) Synapt G2-Si (Waters) using data-independent acquisition (DIA) approach by HDMS^E^ mode (Waters). The tune page for the ionization source was set with the following parameters: 2.60 kV in the sampler capillary, 30 V in the sampling cone, 30 V in the source offset, 70 °C for the source temperature, 0.6 bar for the nano flow gas and 120 L h^−1^ for the purge gas flow. Two chromatograms were acquired (low- and high-energy chromatograms) in positive mode in a range of m/z 50–2000 with a velocity of 0.5 scans s^−1^. No collision energy was applied to obtain the low-energy chromatogram, while for the high-energy chromatograms, the precursor ions were fragmented in the transfer using a collision energy ramp of 19–55 V. Generated raw files containing MS and MS/MS spectra were deconvoluted and compared using ProteinLynx Global SERVER (PLGS) v3.0.3 software Waters [38] against a reversed *Homo sapiens* database (downloaded from Uniprot, 71,785 protein sequences, last modification 1 December 2017). Workflow parameters were as follows: trypsin as a cut enzyme and one missed cleavage allowed; carbamidometh©(C) as a fixed modification and amidation (N-term), deamidation (N,Q), oxidation (M), Phosphoryl (S,T,Y) as variable modifications. Automatic peptide and fragment tolerance, two minimum fragment ion matches per peptide, five minimum fragment ion matches per protein, one minimum peptide match per protein and a false discovery rate (FDR) ≤4%. All identifications had a percentage of reliability ≥95% (Protein AutoCurate green). Synapt G2-Si was calibrated with [Glu^1^]-Fibrinopeptide, [M + 2H]^2+^ = 785.84261 at ≤ 1 ppm.

### 4.5. Isolation of Kidney and HEK293 Cell Mitochondria

Male Wistar rats (200–250 g) were anesthetized by intraperitoneal administration of pentobarbital sodium (50 mg/Kg). Kidney cortex was excised and cooled by immersion in isolation buffer (225 mM D-mannitol, 75 mM sucrose, 1 mM EDTA, 5 mM HEPES, 0.1% fatty acid-free BSA, pH = 7.4) at 4 °C and then cut in small pieces. The isolation procedure was performed at 4 °C. The kidney capsule was removed; tissues were washed with isolation buffer to remove blood, were cut and homogenized with three up/down strokes in a glass Potter-Elvehjem homogenizer with a Teflon@ pestle with 2 mL of isolation buffer. The homogenates were centrifuged at 4000× *g* for 5 min to precipitate debris and unbroken tissue. The supernatant was transferred to new tubes and centrifuged at 12,500× *g* for 15 min without centrifuge brake. The supernatant was recovered as control and the mitochondrial-enriched fraction (bottom) was gently resuspended in 1 mL of BSA-free isolation buffer and centrifuged at 10,000× *g* for 10 min. Finally, the pellet was gently resuspended in 180 μL of BSA-free isolation buffer. Isolation of HEK293 cells mitochondria for immunoblotting was prepared as previously described [39] with minor modifications. Briefly, HEK293 were collected in cold PBS and centrifuged at 700× *g* for 10 min at 4 °C. The cell pellet was resuspended and homogenized in a mitochondrial isolation buffer (MIB; 200 mM sucrose, 10 mM Tris/MOPS pH 7.4, 1 mM EGTA/Tris), using a Teflon pestle attached to a drill at medium speed for three passes. Homogenized cells were passed through two syringes (18 gauge 1 ½ inch and 27 gauge ½ inch). To isolate the mitochondria, the homogenate was centrifuged at 600× *g* for 10 min at 4 °C. The supernatant was transferred to a new tube and centrifuged at 10,000× *g* for 10 min at 4 °C. The mitochondrial pellet was washed 2× with MIB, centrifuged at 10,000× *g* for 10 min at 4 °C, and resuspended with RIPA buffer. For purification of mitochondria for patch-clamp assays, the supernatant obtained from either HEK293T cells or renal cortex was mixed with Percoll to form an 8% (v/v) solution that was stratified with a 15% (v/v) Percoll solution carefully added through the walls. Tubes were centrifuged at 12,500× *g* for 10 min without centrifuge brake. The supernatant was discarded and the mitochondrial-enriched fraction (bottom) was gently resuspended in 1 mL of BSA-free isolation buffer and centrifuged at 10,000× *g* for 10 min. Finally, the pellet was resuspended in 100 μL of BSA-free isolation buffer. All procedures were performed at 4 °C. The protein content was measured by the Lowry method.

### 4.6. Immunoblotting

For SDS-PAGE electrophoresis, the samples were mixed with loading buffer and boiled for 5–8 min. Proteins were separated in 10% acrylamide gels and transferred to 0.2 mm PVDF membrane. Blots were blocked for 1 h at room temperature (RT) in Tris-buffered saline with 0.1% Tween-20 (TBST) containing 5% blotting-grade dry milk, incubated overnight with primary antibody diluted in blocking buffer at 4 °C and washed 3× for 10 min in TBST. Then were incubated with secondary antibody horseradish peroxidase-conjugated for 1h at RT and washed 3× for 10 min in TBST. Dilution of primary antibodies applied to blots from transfected HEK293T cells and isolated mitochondria was 1:1000 for anti-HCN1–3, and 1:1200 for anti-HCN4. As positive control of mitochondrial purification we used polyclonal anti-VDAC and as negative control, anti-α-tubulin. Immunoreactivity was detected using Immobilon Western Chemiluminescent HRP Substrate.

### 4.7. Immunogold Electron Microscopy 

For immunogold labeling, adult Wistar rats were euthanized with intraperitoneal Pentothal, both kidneys were immediately removed, thin tissue slices were obtained and immersed into Karnovsky fixer solution (4% paraformaldehyde and 1.5% glutaraldehyde). Animals were treated in accordance to “Technical specifications for the production, use and care of laboratory animals” (NOM-062-ZOO-1999), and experiments approved by the School of Medicine UNAM, Committee on Animal Research and Ethics (86-2008, date of approval 8 September 2008). The kidney cortex was selected and sectioned in small tissue fragments that were deposited into glass tubes and fixed by immersion in the same fixer solution during 4 h at 20 °C, dehydrated and infiltrated with LR-White hydrosoluble resin. Ultrathin sections of 60–70 nm were placed on nickel grids and incubated overnight at 4 °C with specific anti-HCN-3 (1:100) rabbit antibodies followed by a 2-h incubation at room temperature with goat anti-rabbit IgG conjugated to 5-nm gold particles. The grids were contrasted with uranyl acetate and examined with FEI Tecnai G2 Spirit transmission electron microscope (Hillsboro, OR, USA). The human sample was from a transplant donor and was recovered from formalin-fixed paraffin-embedded tissues; later it was fixed for a day in formaldehyde at 10% in PBS buffer. These conditions did not help to preserve the sample for electron microscopy.

### 4.8. Patch-Clamp Recordings of Whole-Cell and Whole-Mitochondria

HEK293T cells expressing the different constructs described in Figure 2 were placed on coverslips coated with polylysine. Cells were studied between 24–48 h post-transfection. Coverslips were mounted on an open perfusion chamber (TIRF Labs, Cary, NC, USA). The patch clamp amplifier used for whole-cell recordings was the EPC-10 (HEKA Electroniks, Lambrecht Germany). The patch clamp pipettes were prepared from Corning 7052 glass and had a resistance of 5–10 MΩ when filled with the pipette solution (see below). Pipette tip diameter under these conditions was smaller than 1 micron which was sufficiently small to patch large mitochondrion. An Ag/AgCl electrode was utilized to attain electrical continuity and was connected to the bath solution via a KCl-agar bridge. Whole-cell currents were studied in the whole-cell mode with cells expressing the different constructs carrying HCN channels. The pipette solution was: KCl 150 mM, EGTA 5 mM, HEPES 10 mM, pH 7.2. The bath solution contained: KCl 150 mM, glucose 30 mM and HEPES 10 mM, pH adjusted to 7.2. Osmolarity of both solutions was adjusted to 310 mOsm with mannitol. The voltage protocol consisted of 20 mV steps from −120 mV to +60 mV with a holding potential of 0 mV with a duration of 2 s.

For whole-mitochondria currents, mitochondria were isolated either from HEK293 cells expressing the different HCN channels indicated in Figure 2, or from the renal cortex. Isolated mitochondria were identified using fluorescence microscopy to separate mitochondria from cellular debris. Mitochondria were incubated for 3 min with 5 µM of the fluorescent indicator MitoTracker Green FM. Mitochondria were identified under fluorescence microscopy using the emission filter 525/50 nm (peak emission for MitoTracker Green FM is 516 nm). Excitation was produced using a 490 nm, 205 mW LED from Thorlabs (excitation peak for MitoTracker Green FM is 490 nm). Whole-cell and whole-mitochondria currents were imported into Igor pro v. 7 (Wavemetrics, Tigard, OR, USA) for further analysis and plotting. Final figures were created with Adobe Illustrator (Adobe Systems).

### 4.9. Mitochondrial Oxygen Consumption

Mitochondrial oxygen consumption rates were evaluated with a high resolution respirometry (oxygraph O2k, OROBOROS, Innsbruck, Austria) at 37 °C. Isolated mitochondria were loaded into the chamber with 2 mL of MiR05 respiration buffer (0.5 mM EGTA, 3 mM MgCl_2_, 60 mM K-lactobionate, 20 mM taurine, 10 mM KH_2_PO_4_, 20 mM HEPES, 110 mM sucrose and 1 g/L essentially fatty acid free BSA). The mitochondrial electron transfer system (ETS) was started by addition of complex I (CI) linked substrates: 5 mM sodium pyruvate, 2 mM malate and 10 mM glutamate. After stabilization, the corresponding inhibitor (100 µM ZD7288) or vehicle (water) was added and respiration in state 2 (S2) was determined. Respiration in state 3 (S3) was stimulated by the addition of 2.5 mM ADP, later 2.5 μM oligomycin was used to induced state 4 (S4o) respiration. All the above parameters were corrected by residual respiration (ROX), which was obtained by addition of 0.5 µM rotenone (complex I inhibitor) and 2.5 µM antimycin A (complex III inhibitor). The respiratory control index (RCI) was defined as the S3/S4o ratio. All values were normalized by total protein content measured by the Lowry method.

Oxygen consumption in HEK293 cells was performed using 2 mL of culture medium. Each experiment was initiated by addition of the cells. The respiratory parameters were: 1. Routine respiration defined as oxygen consumption by the cells; 2. Leak of the respiration, corresponding to oxygen consumption in the presence of 5 μM oligomycin; 3. Respiratory control index (RCI) corresponding to the ratio basal/leak; 4. Respiration attributable to oxidative phosphorylation (P) was calculated by the formula: Routine minus Leak. All parameters were corrected by subtracting the non-mitochondrial respiration, obtained by the addition of 1 μM rotenone, 5 μM antimycin A, and normalized by the number of cells determined by trypan blue. 

### 4.10. Mitochondrial Membrane Potential (ΔΨm) Monitored by Safranin O and JC-1 Staining

ΔΨm was determined in tandem with the oxygen consumption assays using an O2k-Fluorometer module (OROBOROS). Briefly, the changes in safranin O fluorescence (3 µM) in MiR05 medium were used to monitor changes in ΔΨm. To stimulate complex I linked respiration, 5 mM sodium pyruvate, 2 mM malate and 10 mM glutamate were added. ΔΨm in S2 was obtained after addition of the corresponding inhibitor or vehicle (see above); ΔΨm in S3 was estimated by addition of 2.5 mM ADP and in S4o 2.5 μM oligomycin was added. Finally, 0.5 µM rotenone and 2.5 µM antimycin A were used to completely dissipate ΔΨm and correct by non-specific interactions. Calibration curves of safranin O were employed to ensure the linearity of the assay and the ΔΨm sensibility to CCCP was employed to confirm the veracity of the assay. Results were expressed as the percent of changes in the measurable concentration of safranin O of the sample with respect to the control change. To measure ΔΨm potential by JC-1, 7000 cells were cultured into wells of 96-well plate. Cells were transfected with pcDNA3 (control) or pcDNA3-HCN3 (see above, transfection of HEK cells). After transfection, cells were incubated with 8 μg/mL JC-1 for 60 min on non-supplemented medium. To remove excess probe, cells were washed three times with PBS; fresh supplemented medium was placed on cultures. Depolarized-related (green) fluorescence was measured at 525 nm and polarized-related (red) signal was read at 590 nm, both emissions were obtained at 488 nm excitation. ZD7288 and CCCP were used at 50 µM. Data and representative images were obtained with a Cytation 5 Cell Imaging Multi-Mode reader (BioTek Instruments, Winooski, VT, USA), with GFP and RFP filters. JC-1 fluorescence was calculated as the ratio of red fluorescence and green fluorescence and were normalized with HEK cells (control) without inhibitor. 

## 5. Conclusions

HCN3 constitutes a novel mitochondrial K^+^ channel in the kidney that is coupled to the respiratory chain and ATP synthesis. HCN3 produces a mild uncoupling effect in renal mitochondria and therefore would alter the rate of ROS production and be protective in the kidney. HCN pacemaker channels in the kidney mitochondria contribute importantly to the bioenergetic field and open a new route to the understanding of mitochondrial diseases and new therapeutic avenues. Finally, our study predicts that mitochondria from other organs like heart, brain and liver should also express HCN3 and HCN4 channels.

## Figures and Tables

**Table 1:** List of HCN3 channel-interacting proteins identified by LC/MS/MS during mass spectrometry.

Protein Name	UniProt Accession	UniProt Entry Name	Gene Name	Molecular Mass (kDa)
40S ribosomal protein S3 *	E9PPU1	E9PPU1_HUMAN	*RPS3*	17.4
ATP synthase subunit alpha_ mitochondrial *	P25705	ATPA_HUMAN	*ATP5F1A*	59.9
ATP synthase subunit beta_ mitochondrial *	P06576	ATPB_HUMAN	*ATP5F1B*	56.6
ATP-dependent RNA helicase DDX3X *	A0A2R8Y5G6	A0A2R8Y5G6_HUMAN	*DDX3X*	69.4
C-1-tetrahydrofolate synthase_ cytoplasmic *	P11586	C1TC_HUMAN	*MTHFD1*	102.2
Clathrin heavy chain 2	A0A087WX41	A0A087WX41_HUMAN	*CLTCL1*	144.1
Elongation factor Tu_ mitochondrial *	P49411	EFTU_HUMAN	*TUFM*	49.9
Eukaryotic translation initiation factor 4 gamma 1	Q04637	IF4G1_HUMAN	*EIF4G1*	176.2
Fatty acid synthase	A0A0U1RQF0	A0A0U1RQF0_HUMAN	*FASN*	275.8
Filamin-A	A0A087WWY3	A0A087WWY3_HUMAN	*FLNA*	248.3
Golgin subfamily B member 1	Q14789	GOGB1_HUMAN	*GOLGB1*	377.4
Heat shock 70 kDa protein 1A *	P0DMV8	HS71A_HUMAN	*HSPA1A*	70.3
Heat shock-related 70 kDa protein 2	P54652	HSP72_HUMAN	*HSPA2*	70.3
Isoform 2 of Clathrin heavy chain 1	Q00610-2	CLH1_HUMAN	*CLTC*	189.7
Isoform 2 of Glyceraldehyde-3-phosphate dehydrogenase	P04406-2	G3P_HUMAN	*GAPDH*	31.7
Isoform 2 of Heat shock protein 75 kDa_ mitochondrial *	Q12931-2	TRAP1_HUMAN	*TRAP1*	74.5
Isoform 3 of Pyruvate kinase PKM	P14618-3	KPYM_HUMAN	*PKM*	56.9
Isoform 3 of Vacuolar protein sorting-associated protein 13C	Q709C8-3	VP13C_HUMAN	*VPS13C*	419.6
Isoform MBP-1 of Alpha-enolase	P06733-2	ENOA_HUMAN	*ENO1*	37.3
Isoform Short of Delta-1-pyrroline-5-carboxylate synthase *	P54886-2	P5CS_HUMAN	*ALDH18A1*	87.8
Matrix-remodeling-associated protein 5	Q9NR99	MXRA5_HUMAN	*MXRA5*	314.3
Polyadenylate-binding protein 4	Q13310	PABP4_HUMAN	*PABPC4*	71.1
Polyubiquitin-B (Fragment) *	J3QS39	J3QS39_HUMAN	*UBB*	10.5
Prohibitin (Fragment) *	C9JZ20	C9JZ20_HUMAN	*PHB*	22.3
Prohibitin-2 *	Q99623	PHB2_HUMAN	*PHB2*	33.3
Receptor of activated protein C kinase 1	P63244	RACK1_HUMAN	*RACK1*	35.5
RuvB-like 2	Q9Y230	RUVB2_HUMAN	*RUVBL2*	51.3
Tubulin beta chain	Q5JP53	Q5JP53_HUMAN	*TUBB*	48.2
Tubulin beta-4B chain	P68371	TBB4B_HUMAN	*TUBB4B*	50.3
Vimentin	P08670	VIME_HUMAN	*VIM*	53.7

The resulting candidate proteins had a percentage ≥95% of reliability (Protein AutoCurate green), highlighting the robustness of the analytical method. * Proteins identified with at least one Gene Ontology (GO) term related with mitochondria (www.geneontology.org, www.pantherdb.org and www.string-db.org) and these proteins are listed in the actual version of the MitoProteome database (www.mitoproteome.org).

## Abbreviations

IMM	inner mitochondrial membrane
ΔΨm	mitochondrial membrane potential
